# Supplementary material for: Comparative genomics provides new insights into the diversity, physiology, and sexuality of the only industrially exploited tremellomycete: Phaffia rhodozyma
Source: BMC Genomics. 2016 Nov 9;17:901. doi: 10.1186/s12864-016-3244-7 (PMC5103461; doi:10.1186/s12864-016-3244-7)
Supplement: Additional file 6: — List of orphan genes with links to PFAM (related to Additional file 1: Table S1). (ZIP 1428 kb) [file 12864_2016_3244_MOESM6_ESM.zip › PFAM_UNIQUE/G03713_P.html]

#                                                                            --- full sequence --- -------------- this domain -------------   hmm coord   ali coord   env coord
  
# target name        accession   tlen query name           accession   qlen   E-value  score  bias   #  of  c-Evalue  i-Evalue  score  bias  from    to  from    to  from    to  acc description of target
  
#------------------- ---------- ----- -------------------- ---------- ----- --------- ------ ----- --- --- --------- --------- ------ ----- ----- ----- ----- ----- ----- ----- ---- ---------------------
  
G03713\_P             -            539 BicD                 PF09730.4    717       4.6    4.1  11.6   1   2      0.14        32    1.3   4.4   386   426    12    44     1   151 0.60 - Microtubule-associated protein Bicaudal-D  
G03713\_P             -            539 BicD                 PF09730.4    717       4.6    4.1  11.6   2   2    0.0021      0.49    7.3   0.7    89   149   216   273   189   281 0.66 - Microtubule-associated protein Bicaudal-D  
G03713\_P             -            539 Draxin               PF15550.1    324       6.8    4.9  19.8   1   2    0.0022       2.2    6.5  10.2    83   154    10    82     1   147 0.45 - Draxin  
G03713\_P             -            539 Draxin               PF15550.1    324       6.8    4.9  19.8   2   2     0.018        18    3.5   2.0     2    64   227   288   226   306 0.83 - Draxin  
G03713\_P             -            539 DUF904               PF06005.7     72    0.0014   17.6   6.8   1   4        10     6e+03   -7.7  18.3    34    63    14    43     3    44 0.67 - Protein of unknown function (DUF904)  
G03713\_P             -            539 DUF904               PF06005.7     72    0.0014   17.6   6.8   2   4   2.3e-06    0.0014   17.6   6.8     8    53   200   254   198   275 0.81 - Protein of unknown function (DUF904)  
G03713\_P             -            539 DUF904               PF06005.7     72    0.0014   17.6   6.8   3   4       4.1   2.5e+03   -2.4   0.2    39    58   430   449   427   452 0.72 - Protein of unknown function (DUF904)  
G03713\_P             -            539 DUF904               PF06005.7     72    0.0014   17.6   6.8   4   4        10     6e+03   -3.9   0.2    29    46   514   530   510   532 0.49 - Protein of unknown function (DUF904)  
G03713\_P             -            539 DUF972               PF06156.8    107     0.011   14.7   2.9   1   1   4.8e-05     0.011   14.7   2.9    12    83   200   276   195   278 0.79 - Protein of unknown function (DUF972)
